# Nanostructured Lipid Microparticles as Tobramycin Carriers for Pulmonary Drug Delivery

**DOI:** 10.3390/pharmaceutics18091061

**Published:** 2026-08-26

**Authors:** Katarzyna Reczyńska-Kolman, Jan Grabiński, Konrad Kwiecień, Dorota Ochońska, Kinga Pielichowska, Monika Brzychczy-Włoch, Elżbieta Pamuła

**Affiliations:** 1 Department of Biomaterials and Composites, Faculty of Materials Science and Ceramics, AGH University of Krakow, Al. Mickiewicza 30, 30-059 Kraków, Poland; 2 Faculty of Electrical Engineering, Automatics, Computer Science and Biomedical Engineering, AGH University of Krakow, Al. Mickiewicza 30, 30-059 Kraków, Poland; 3 Department of Molecular Medical Microbiology, Chair of Microbiology, Faculty of Medicine, Jagiellonian University Medical College, 18 Czysta Street, 31-121 Kraków, Poland; 4 Department of Glass Technology and Amorphous Coatings, Faculty of Materials Science and Ceramics, AGH University of Krakow, Al. Mickiewicza 30, 30-059 Kraków, Poland

**Keywords:** nanostructured lipid carriers, lower respiratory tract infections, microparticles, fatty acids, tobramycin

## Abstract

**Background/Objectives:** Targeted pulmonary delivery of antibiotics offers a favorable approach for the treatment of lower respiratory tract infections (LRTIs) by enhancing local drug concentrations while minimizing systemic exposure. This study aimed to develop and characterize fatty acid-based nanostructured lipid carrier (NLC) microparticles, including tobramycin-loaded formulations, and assess their potential application as carriers for pulmonary drug delivery. **Methods:** Tobramycin-loaded NLC microparticles were prepared using a hot emulsification method, with lauric acid as the solid lipid and oleic acid as the liquid lipid. The obtained formulations were characterized in terms of morphology, particle size distribution, zeta potential, thermal properties, encapsulation efficiency, drug-release behavior, aerodynamic properties, and powder flowability. Antibacterial activity was evaluated against *S. aureus* and *P. aeruginosa*, while cytocompatibility was assessed using BEAS-2B human-lung epithelial cells. **Results:** The developed microparticles exhibited spherical morphology, with particle characteristics influenced by lipid composition. Incorporation of oleic acid increased tobramycin encapsulation efficiency, reaching approximately 75% at liquid lipid concentrations of 5–15%. Theoretical aerodynamic diameters remained within the respirable range, and increasing oleic acid content improved powder flowability. Drug-release behavior was strongly dependent on lipid composition, with the optimized formulation providing prolonged tobramycin release over 72 h. All formulations preserved antibacterial activity against *S. aureus* and *P. aeruginosa* and demonstrated good cytocompatibility with BEAS-2B cells. **Conclusions:** Fatty acid-based nanostructured lipid microparticles represent a promising active carrier platform for the pulmonary delivery of tobramycin. The developed system demonstrated efficient drug encapsulation, satisfactory particle characteristics, controlled drug release, preserved antibacterial activity, and good biocompatibility, making them a suitable component of dry powders for inhalation (DPI).

## 1. Introduction

Lower respiratory tract infections (LRTIs) remain the world’s deadliest communicable disease after COVID-19, accounting for approximately 2.5 million deaths in 2021, according to the World Health Organization [1]. LRTIs continue to impose a substantial global health burden, with approximately 2.5 million LRTI-related deaths estimated for 2023 [2]. A variety of pathogens, including bacterial, viral, and fungal agents, contribute to LRTIs, with bacterial infections accounting for the majority of related mortality [3]. Notably, *Streptococcus pneumoniae* is the leading cause of pneumonia worldwide, and together with other bacterial pathogens, such as *Staphylococcus aureus*, *Klebsiella pneumoniae*, and *Pseudomonas aeruginosa*, it represents a significant contributor to LRTI-related deaths across all age groups [3,4]. Accordingly, treatment strategies primarily rely on antibiotic therapy, administered via oral, intravenous, or pulmonary routes, involving either empirical broad-spectrum or targeted agents depending on the identified pathogen [4].

Pulmonary administration offers a wide range of advantages; however, this administration mode requires a drug to be processed into a suitable formulation [5,6]. Particle deposition in the respiratory tract depends on physiological factors like lung morphology, inhalation technique, and device design, as well as formulation factors like aerosol performance, particle size, morphology, and stability [7,8]. High encapsulation efficacy of the drug is necessary to achieve high concentration of the drug at the site of action, while all additional excipient have to be non-toxic and biodegradable and cannot pose additional burden to the lungs [9]. While physiological factors are hard to modulate, optimizing formulation properties can enhance efficacy by adjusting particle size, morphology, aerodynamic properties, and surface modifications.

Aerodynamic properties are crucial for inhalable-drug delivery. The aerodynamic diameter of a particle, which determines its deposition in the respiratory tract, can differ significantly from its physical diameter. The optimal particle size for inhalation is generally 1–5 µm; particles larger than 5 µm deposit in the upper airways, while those smaller may be exhaled. Particles larger than 2 µm tend to deposit in the bronchi, while smaller particles reach the alveoli [5,7,10].

Lipid microparticles are advantageous as drug carriers due to their biodegradability, cost-effective production, stability, and controlled-release properties. What is more important, various lipids, such as fatty acids or triglycerides, occur naturally in human bodies, and they can be easily degraded and metabolized by the cells. For pulmonary applications, they offer good aerosolization properties and the ability to be converted into dry powder formulations [11]. Additionally, their low density reduces inter-particle forces, improving aerosol-delivery efficiency, and their properties can be optimized through manufacturing processes to enhance performance [12].

One of the biggest disadvantages of conventional lipid microparticles is their highly crystalline and usually hydrophobic character, which limits their ability for efficient encapsulation of water-soluble drugs. Nanostructured lipid carriers (NLCs) were developed to overcome this issue. This concept is based on the assumption that the addition of liquid lipid to solid lipid matrix of the particles distorts the internal arrangement of molecules inside NLC particles, resulting in a less organized lipid core. This increases the drug-loading capacity of NLCs compared to particles made solely of solid lipids [13,14,15]. The majority of NLC-based formulations described in the literature focus on the development of nanoparticle systems. For example, itraconazole-loaded nanostructured lipid carrier nanoparticles have been developed for pulmonary administration in the context of pulmonary aspergillosis [16]. Similarly, mannose-functionalized isoniazid-loaded NLC nanoparticles have been reported for lung delivery in tuberculosis treatment [17]. More recently, magnolol-loaded NLC nanoparticles have been described for pulmonary delivery in chronic respiratory diseases such as chronic obstructive pulmonary disease (COPD) [18]. In contrast, NLC-based microparticle systems remain comparatively underexplored and therefore warrant further investigation as potential pulmonary drug delivery systems.

While lipid-based pulmonary formulations of tobramycin have previously been reported, including NLCs designed to improve pulmonary delivery in cystic fibrosis [19], the present study explores a novel formulation rationale aimed at simultaneously improving formulation properties and providing a foundation for future multifunctional carrier systems for pulmonary delivery. Both proposed matrix components were intentionally chosen based on their reported biological activities. Lauric acid has been shown to possess intrinsic antimicrobial activity, whereas oleic acid has attracted increasing attention not only as a liquid lipid capable of reducing lipid crystallinity and enhancing drug incorporation, but also as a compound exhibiting antibacterial effects and potential synergistic and hydrophobic ion-pairing interactions with aminoglycoside antibiotics [20,21,22].

The aim of this study was to develop and characterize fatty acid-based NLC microparticles as active carrier systems with potential for the pulmonary delivery of tobramycin, which may serve as a dry powder for inhalation (DPI) components. Natural fatty acids were used as matrix materials: lauric acid (C12:0) was used as a solid lipid, while oleic acid (C18:1) was used as a liquid lipid. Tobramycin was selected as a model drug for encapsulation, as it is highly active against both Gram-positive and Gram-negative bacteria; however, its high water solubility and several side effects associated with systemic administration limit its use [23].

The research questions asked were:(1) whether NLC technology could be applied to fabricate microparticles with diameters in the range of 1 to 5 µm; (2) whether the incorporation of a liquid lipid could indeed increase the tobramycin loading capacity; and (3) whether the obtained NLC microparticles would exhibit physicochemical and biological properties supportive of their potential use as carriers in pulmonary drug delivery, including antibacterial activity against common bacterial strains that cause respiratory tract infections.

## 2. Materials and Methods

### 2.1. Reagents

Lauric acid (C12:0), poly(vinyl alcohol) (Mowiol 4-88, 31 kDa), tobramycin sulfate, *o*-phthalaldehyde (OPA), 2-mercaptoethanol, boric acid, Triton X-100, cytotoxicity detection kit (lactate dehydrogenase activity based, LDH), resazurin sodium salt (AlamarBlue reagent, 0.1 mg/mL in phosphate buffered saline), and crystal violet (0.25% *w*/*v* in 20% methanol) were purchased from Sigma Aldrich (Steinheim, Germany). Oleic acid (C18:1) was supplied by Lipoid GmbH (Ludwigshafen, Germany). Methanol was obtained from Avantor Performance Materials (Gliwice, Poland).

Gamble solution was prepared based on [24], using magnesium chloride (0.095 g/L), sodium chloride (6.019 g/L), potassium chloride (0.298 g/L), disodium hydrogen phosphate (0.126 g/L), sodium sulfate (0.063 g/L), calcium chloride (0.277 g/L), sodium acetate (0.574 g/L), sodium hydrogen carbonate (2.604 g/L), and sodium citrate dihydrate (0.097 g/L). All reagents were obtained from Avantor Performance Materials (Gliwice, Poland).

BEAS-2B human non-malignant cells (CRL-3588), and bacterial strains *Staphylococcus aureus* (ATCC 25923), *Klebsiella pneumoniae* (ATCC 700603), *Pseudomonas aeruginosa* (ATCC 27853), and *Escherichia coli* (ATCC 25922) were purchased from American Type Culture Collection (ATCC, Manassas, Virginia, USA). For cell cultures, Dulbecco’s Modified Eagle Medium supplemented with 10% fetal bovine serum and 1% penicillin/streptomycin (PAN Biotech, Aidenbach, Germany) was used. Phosphate-buffered saline (PBS) was obtained from VWR Chemicals (Gdańsk, Poland).

### 2.2. Fabrication of Unloaded Microparticles

Unloaded nanostructured lipid carriers were fabricated using the hot oil-in-water emulsification method, with their final composition listed in Table 1. Briefly, 200 mg of lipid mixture (consisting of lauric acid only for 0% OLE samples or a mixture of lauric acid with 5%, 10%, or 15% *w*/*w* addition of oleic acid) was heated to 65 °C using a water bath. For each batch of microparticles, 2 mL of 10% *w*/*v* PVA solution was also heated to 65 °C in separate Falcon tubes (15 mL volume). Melted lipids were transferred to PVA solution, emulsified using a vortex mixer for 90 s, and immediately cooled down by pouring into 20–30 mL of liquid nitrogen. Thawed microparticle suspension was purified via repeated centrifugation (18,000 rpm, 30 min, 4 °C, MPW Instruments, Warsaw, Poland) and rinsing with Milli-Q water. The obtained microparticles were frozen again using liquid nitrogen, freeze-dried for 24 h (Alpha 1-2 LD, Christ, Osterode am Harz, Germany), and then stored at −20 °C until further use.

### 2.3. Characterization of Unloaded Microparticles

Unloaded microparticles were characterized in terms of microstructure and particle size using optical microscopy, followed by preliminary in vitro evaluation in contact with BEAS-2B lung epithelial cells.

#### 2.3.1. Morphology

Approximately 1 mg of freeze-dried microparticles was resuspended in 1 mL of Milli-Q water. Then, 10 µL of the suspension was placed on a glass slide and covered with a glass slip. For each sample, at least 3 images were taken using an optical microscope (Axiovert 40 CFL inverted microscope, Zeiss, Jena, Germany, or MB200, OPTA-TECH, Warsaw, Poland) at a magnification of 600×. The diameters of at least 700 microparticles were measured for each sample using ImageJ software (ij154-win-java8 version, National Institutes of Health, Bethesda, MD, USA).

#### 2.3.2. Cytotoxicity

BEAS-2B normal lung epithelial cells were used for preliminary evaluation of the cytotoxicity of unloaded microparticles. A total of 10,000 cells were seeded in a 96-well plate in 100 µL of complete cell culture medium (DMEM supplemented with 10% FBS and 1% penicillin/streptomycin) and allowed to proliferate overnight. The next day, cell culture medium was replaced with 100 µL of microparticle suspension at a concentration of 100 µg/mL (the microparticles were previously sterilized using UV irradiation for 25 min). After 24 h of treatment, 50 µL of culture medium was transferred to a transparent 96-well plate and mixed with 50 µL of LDH reagent (prepared as indicated by the manufacturer). After 20 min of incubation at room temperature in the dark, the absorbance was measured at 492 nm (FluoStar Omega, BMG Labtech, Ortenberg, Germany) for LDH release determination. The remaining medium was withdrawn from the wells and replaced with 150 µL of culture medium containing 5% vol. AlamarBlue reagent. After 3 h of incubation at 37 °C, 100 µL of the medium was transferred to a black 96-well plate, and the fluorescence was measured at λ_ex_ = 530 nm and λ_em_ = 590 nm (FLUOstar Omega, BMG Labtech). Percentage resazurin reduction was calculated according to Equation (1):

(1) $$Resazurin\;reduction\;\left(\%\right)=\frac{F_{x}-F_{0\%}}{F_{100\%}-F_{0\%}}\times 100\%\;,$$

where *F_x_* is the fluorescence of the sample, *F*_0%_ is the fluorescence of the medium with AlamarBlue reagent without cells, and *F*_100%_ is the fluorescence of the completely reduced reagent (medium with the reagent autoclaved for 15 min at 121 °C).

The cells cultured in complete cell culture medium were used as a negative control, while for a positive control, the cells were treated with Triton-X 100 (1% vol. solution in cell culture medium) for 20 min before the assays.

#### 2.3.3. Particle Size Distribution and Zeta Potential

The size distribution of the unloaded microparticles was determined by laser diffraction using a Mastersizer 3000E (Malvern Panalytical, Malvern, UK), and the zeta potential was measured by dynamic light scattering using a Zetasizer (Malvern Panalytical, Malvern, UK). All measurements were performed in triplicate.

#### 2.3.4. Differential Scanning Calorimetry (DSC)

The thermal properties of the samples were analyzed using a DSC 250 Discovery differential scanning calorimeter (TA Instruments, New Castle, DE, USA). The instrument was calibrated using indium and zinc standards. Measurements were carried out over a temperature range of −50 to 80 °C (lauric acid, oleic acid, and unloaded NLC microparticles), at heating and cooling rates of 10 °C/min under a nitrogen atmosphere with a flow rate of 30 mL/min. Samples with a mass of approximately 1.5 mg were sealed in aluminum pans prior to analysis.

### 2.4. Fabrication of Tobramycin-Loaded Microparticles

Tobramycin-loaded nanostructured lipid carriers were fabricated using the modified hot water-in-oil-in-water emulsification method, with their final composition listed in Table 2. Briefly, 20 mg of tobramycin was gently dissolved in 50 µL of Milli-Q water and emulsified into lipid phase (180 mg of lipid mixture consisting of lauric acid only for 0% OLE samples or a mixture of lauric acid with 5%, 10%, or 15% *w*/*w* addition of oleic acid) melted at 65 °C using an ultrasonic homogenizer (1 min, 40% amplitude, VibraCell, Sonics, Newtown, CT, USA). As-obtained primary emulsion was transferred to external aqueous phase (2 mL of aqueous PVA solution at a concentration of 10% *w*/*v*) and processed in the same way as in the case of unloaded microparticles. The supernatant left after the first centrifugation of the microparticles was collected and used for the determination of the encapsulation efficacy.

### 2.5. Characterization of Tobramycin-Loaded Microparticles

For drug-loaded microparticles, the characterization of morphology, size distribution, zeta potential, and differential scanning calorimetry was performed analogically, as described in Section 2.4. Only the DSC measurement was performed here, over the temperature range of −50 to 220 °C.

#### 2.5.1. Composition

Encapsulation efficacy (*EE*) was measured indirectly using supernatants left after microparticle purification. The concentration of tobramycin in the supernatants was measured using an *o*-phthalaldehyde (OPA)-based amine detection method. In total, 6 mg of OPA was dissolved in 100 µL of methanol and 20 µL of 2-mercaptoethanol and diluted with 10 mL of 400 mM boric acid solution (pH = 10.4). Then, 50 µL of the prepared reaction mixture was combined with 50 µL of the supernatant sample in a 96-well black plate. After 10 min of incubation at room temperature and in the dark, the fluorescence intensity was measured at λ_ex_ = 340 nm and λ_em_ = 450 nm. LL-37 EE and drug loading (*DL*) were calculated according to Equations (2) and (3), respectively:

(2) $$EE\;\left(\%\right)=\frac{mass\;of\;tobramycin\;in\;microparticles}{initial\;mass\;of\;tobramycin}\times 100\%\;,$$

(3) $$DL\;\left(\%\right)=\frac{mass\;of\;tobramycin\;in\;microparticles}{mass\;of\;microparticles}\times 100\%\;.$$

*EE* and *DL* were determined in triplicate for at least two independent batches of microparticles.

#### 2.5.2. Fourier-Transform Infrared Spectroscopy (FTIR)

FTIR analysis of pure lauric and oleic acids, and tobramycin and tobramycin-loaded nanostructured lipid microparticles was performed (Bruker Tensor 27 spectrophotometer, Bruker Daltonics, Bremen, Germany) in ATR mode with the use of diamond/ZnSe crystal. The spectra were recorded between 4000 and 400 cm^−1^, at a 4 cm^−1^ resolution. For each sample, 64 single scans were collected. Obtained spectra were analyzed using OPUS software (Bruker Optics, Ettlingen, Germany).

#### 2.5.3. Tobramycin Release

In vitro tobramycin release was estimated by the membrane diffusion method, using Gamble solution as acceptor liquid. Approximately 5 mg of the microparticles (0% OLE + tob, 5% OLE + tob, and 10% OLE + tob) was suspended in 200 µL of Gamble solution, transferred to a membrane pocket (ZelluTrans, MWCO = 10–12 kDa, Roth, Karlsruhe, Germany), and sealed. The membrane was then immersed in 7 mL of Gamble solution and placed at 37 °C on a horizontal shaker (50 rpm). At predetermined time points (i.e., 0.5 h, 1 h, 2 h, 4 h, 8 h, 24 h, 48 h, and 72 h), acceptor solution was transferred to a separate tube and kept at −20 °C for further analysis. A fresh portion of Gamble solution was added to each tube, and the incubation was continued.

Tobramycin content in Gamble solution was measured using the OPA-based amine detection method in the same way as described before. Pure tobramycin (tob) was used as a control. The experiment was run in triplicate for each type of microparticle. The obtained release profiles were further analyzed using KinetDS software (3.0 version, Jagiellonian University, Kraków, Poland) to evaluate the release kinetics and determine the most appropriate mathematical model among zero-order, first-order, second-order, and Korsmeyer–Peppas-with-lag-time models.

#### 2.5.4. Aerodynamic Properties

The bulk density of the microparticles was estimated as described before [25]. The microparticles were loaded into a 1.5 mL polypropylene vial, and their mass and volume were accurately measured. Then, the vial was tapped 100 times on the desk, and the tapped volume of the microparticles was measured. The bulk density was calculated as the mass divided by the volume of powder before tapping, while the tapped density was calculated as the mass divided by the volume of powder after tapping.

The theoretical aerodynamic diameter was calculated according to Equation (4):

(4) $$d_{aero}=d_{g}\sqrt{\frac{\rho_{tapped}}{\chi \cdot \rho_{0}}}\;,$$

where *d_aero_* is the theoretical aerodynamic diameter, *d_g_* is the geometric diameter measured based on microscopy images of the microparticles, χ is the shape factor (for spherical microparticles is equal to 1), *ρ_tapped_* is the tapped density of the microparticles, and *ρ*_0_ is the reference density equal to 1 g/cm^3^.

The Carr index (CI) and Hausner ratio (HR) were calculated as follows, Equations (5) and (6), respectively:

(5) $$CI=\;\frac{\rho_{tapped}-\rho_{bulk}}{\rho_{tapped}}\cdot 100\%\;\;,$$

(6) $$HR=\frac{\rho_{tapped}}{\rho_{bulk}}\;\;,$$

where *ρ_tapped_* is the tapped density of the microparticles, and *ρ_bulk_* is the bulk density of the microparticles.

#### 2.5.5. Antibacterial Properties

The antibacterial activity of the microparticles was assessed by the Kirby–Bauer method (agar diffusion tests) using *Staphylococcus aureus* (ATCC 25923), *Klebsiella pneumoniae* (ATCC 700603), *Pseudomonas aeruginosa* (ATCC 27853), and *Escherichia coli* (ATCC 25922); all strains came from American Type Culture Collection, Manassas, VA, USA. The minimum inhibitory concentrations (MICs) of free tobramycin for all bacterial strains were determined prior to the experiments, using Etest strips (bioMérieux, Marcy-l’Étoile, France) according to the manufacturer’s instructions.

The bacterial suspensions were prepared in PBS at a concentration of 0.5 McFarland (1.5 × 10^8^ CFU/mL), and the bacteria were seeded on tryptic soy agar using a sterile cotton swab. Tobramycin-loaded microparticles were accurately weighted and suspended in PBS at a concentration of 0.2 mg/mL, 1 mg/mL, and 5 mg/mL. Three holes (6 mm in diameter) were cut in each plate and filled with 80 µL of microparticle suspension. PBS was used as a negative control sample, while reference tobramycin discs (10 µg) that were placed on top of the agar were used as a positive control. The plates were kept at 4 °C for 4 h to allow initial diffusion of tobramycin into agar, and then they were transferred to 37 °C for 18 h. After that, the diameter of inhibition of bacterial growth was measured, and optical images of the plates were taken. The experiment was run in triplicate for each sample.

Subsequently, time-kill kinetics were determined for three previously used bacterial strains: *S. aureus*, *P. aeruginosa*, and *E. coli*. *K. pneumoniae* was not included in the time-kill assay, because no antibacterial activity was observed in the preliminary Kirby–Bauer test. The tested antimicrobial agents included tobramycin-loaded lipid microparticles containing 0%, 5%, or 10% oleic acid (0% OLE, 5% OLE, and 10% OLE, respectively) suspended in PBS at a concentration of 1 mg/mL (concentration chosen based on Kirby–Bauer study) and free tobramycin (TB0, TB5, and TB10) in the same dose, as in the particles calculated based on the assessed drug loadings.

Overnight bacterial cultures in the logarithmic growth phase were adjusted to 0.5 McFarland standard and mixed with Mueller–Hinton Broth II (MHB II, Merck, Germany) containing the tested formulations (800 µL bacterial suspension with 100 µL of the tested formulation). The final bacterial inoculum was adjusted to 5 × 10^5^ CFU/mL for *S. aureus* and 1 × 10^6^ CFU/mL for both *P. aeruginosa* and *E. coli*. The resulting suspensions were incubated at 35 °C, with shaking (ES-20/60, Biosan, Riga, Latvia).

At 0, 2, 4, and 24 h, 100 µL aliquots were collected from each tube, serially diluted in sterile 0.9% sodium chloride solution, and 100 µL of the appropriate dilutions were spread onto tryptic soy agar (TSA) plates. Following 24 h of incubation at 37 °C, bacterial colonies were counted using a colony counter (090-LKB 2002, POL-EKO, Wodzisław Śląski, Poland). Time-kill graphs were constructed by plotting the mean bacterial counts (log_10_ CFU/mL) against time to compare the bactericidal activity of free tobramycin and tobramycin released from the lipid microparticles over a 24 h period. Bactericidal activity was defined as a ≥3 log_10_ CFU/mL (99.9%) reduction in viable bacterial counts relative to the initial inoculum, according to CLSI guidelines and the criteria described by Díez-Aguilar et al. [26].

#### 2.5.6. Cytotoxicity

The cytotoxicity of the tobramycin-loaded microparticles was evaluated in the same manner as in the case of unloaded microparticles. Besides the measurements of LDH release and the metabolic activity of the cells, cell morphology was also assessed using crystal violet staining. Once the AlamarBlue assay was finished, the cells were gently rinsed with PBS (3 × 100 µL) and fixed for 20 min with ice-cold 3.8% formaldehyde. Upon formaldehyde withdrawal and rinsing with PBS (1 × 100 µL), the cells were stained with 0.25% crystal violet solution for 10 min at room temperature. After several rounds of rinsing with water, the cells were visualized using an inverted microscope at 100× magnification (Axiovert 40 CFL, Zeiss, Jena, Germany).

### 2.6. Statistical Analyses

Statistical analyses of obtained data were done using a one-way analysis of variance (one-way ANOVA), followed by Tuckey’s post hoc test. The assumptions of normal distribution and equal variance were verified using the Shapiro–Wilk test and Levene median test, respectively (*p*-value > 0.05). The analyses were performed using OriginPro2024 software. The results were presented as mean ± standard deviation (SD). In the case of particle size distribution graphs, box and whiskers feature interquartile range (IQR) (with median marked as a line inside) and the range within 1.5 QR (quartile range); mean values are presented as circles, while dots represent outliers.

## 3. Results

This study focused on the development of nanostructured lipid microparticles for pulmonary delivery of tobramycin. The first part of the study was devoted to fabrication and preliminary characterization of morphology and cytotoxicity of unloaded microparticles differing in the content of liquid lipid (oleic acid, OLE) in the lipid matrix of the particles. Then, tobramycin was added to the microparticles, and the microparticles were evaluated in detail in terms of their morphology, composition, flow characteristics, antibacterial properties, and cytotoxicity.

### 3.1. Unloaded Microparticles

**Figure 1:**
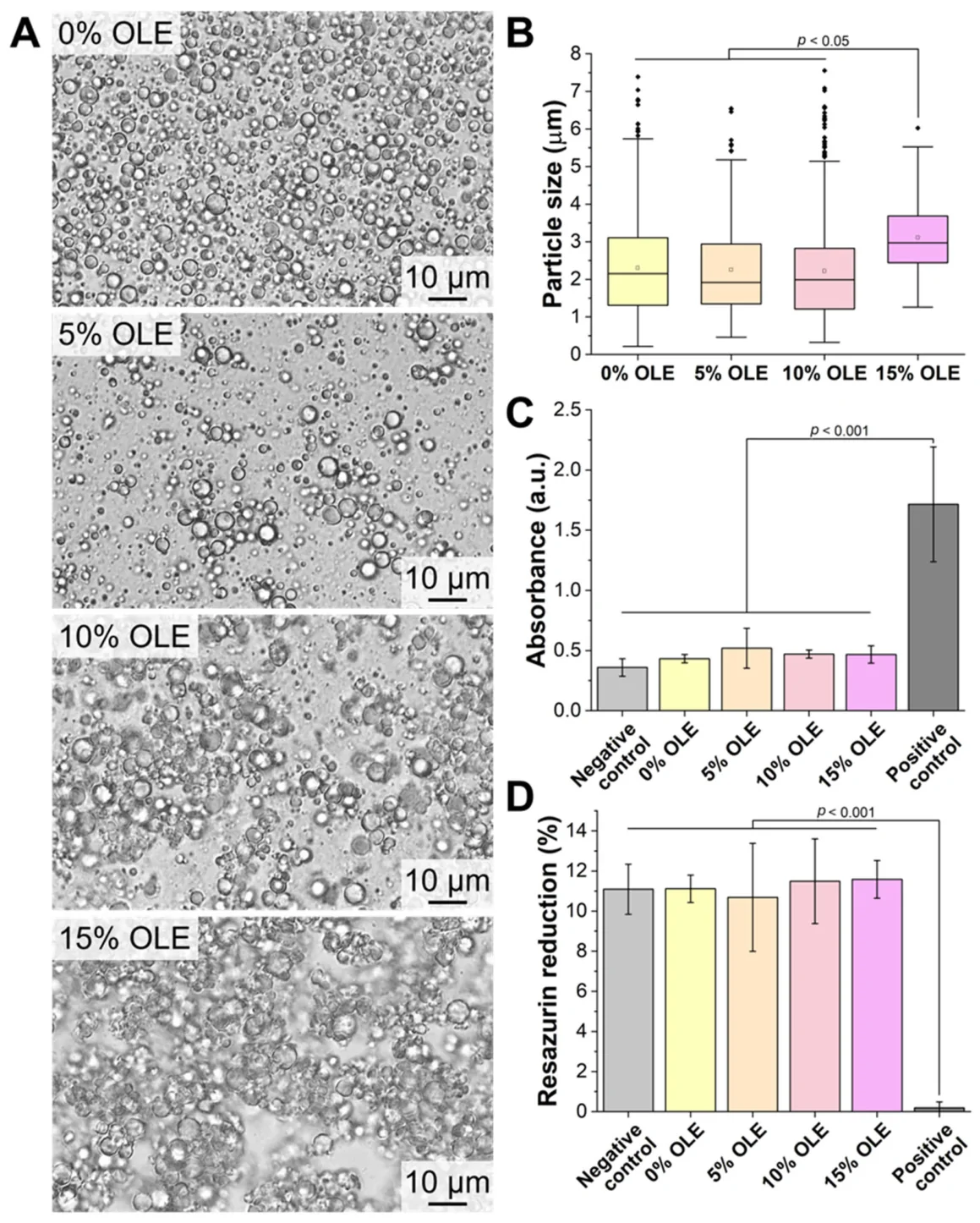
Morphology determined using optical microscopy (**A**), particle size (**B**), and the results of cytotoxicity assays performed on BEAS-2B lung epithelial cells (LDH release (**C**) and metabolic activity assay (**D**)) of unloaded nanostructured lipid carrier (NLC) microparticles.

The unloaded nanostructured lipid microparticles were spherical in shape (Figure 1A), with median diameters of approximately 2 µm, except from 15% OLE microparticles, which were characterized by a significantly larger median diameter equal to 3 µm (Figure 1B). It was also the only sample in which a pronounced agglomeration of the microparticles was observed.

All microparticles were cytocompatible with BEAS-2B non-malignant lung epithelial cells, as evidenced by both LDH (Figure 1C) and metabolic activity (Figure 1D) assays. No statistically significant differences were observed between the microparticles differing in OLE content; thus, all tested microparticles were used in further studies on tobramycin encapsulation.

**Figure 2:**
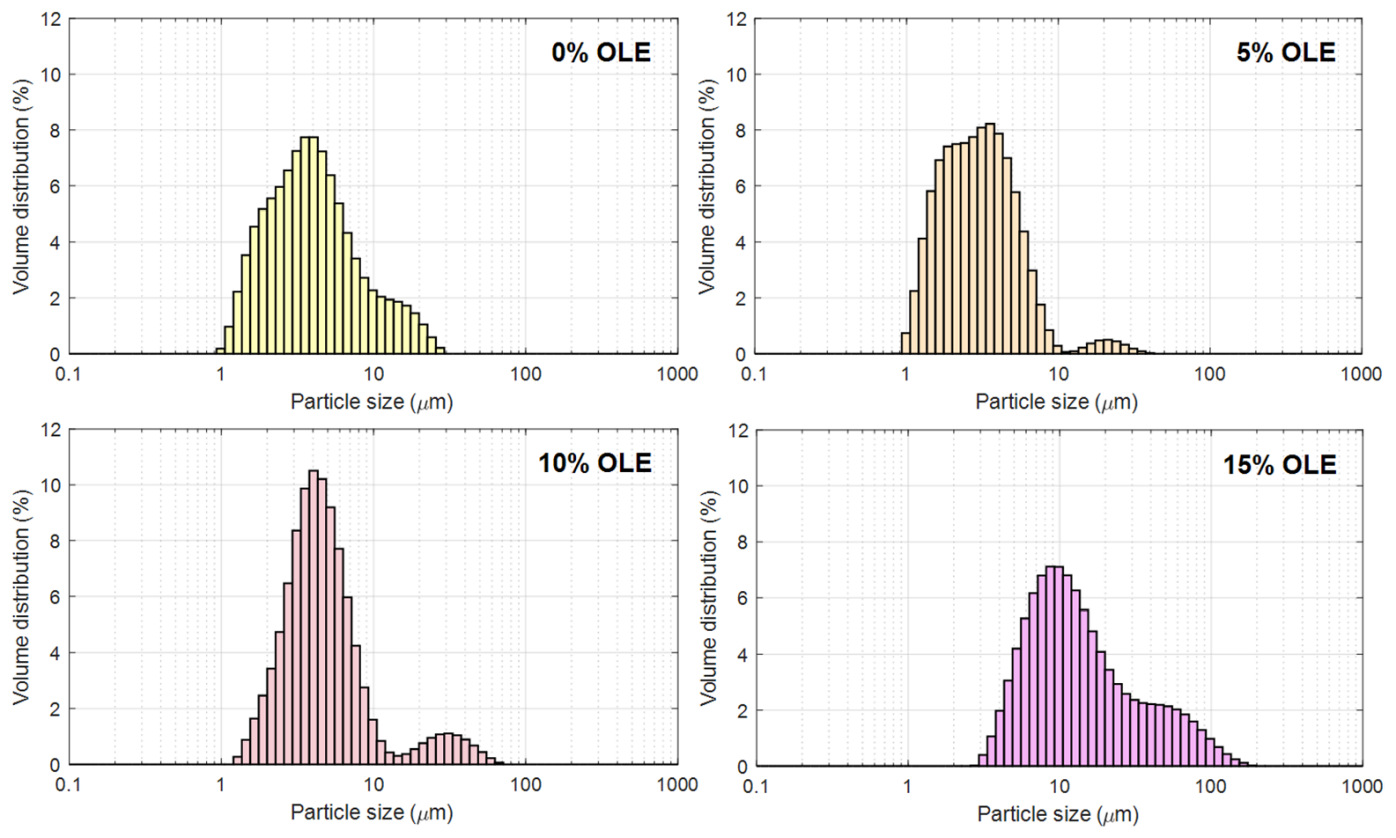
Volume-based particle size distributions of the unloaded microparticles determined by laser diffraction.

The particle size distributions of the unloaded microparticles are presented as volume-weighted distributions (Figure 2). For formulations containing 0%, 5%, and 10% OLE, the highest contribution to the total particle volume was observed within the 1–10 μm size range, with median particle sizes (Dx(50)) of 4.05 μm, 3.12 μm, and 4.66 μm, respectively (Table 3). Increasing the OLE content to 15% resulted in a shift of the distribution toward larger particle sizes, with a higher contribution of particles above 10 μm to the overall volume distribution and an increased median particle size (Dx(50)) of 12.7 μm. The zeta-potential values of the unloaded microparticles ranged from approximately −14 to −19 mV, with no pronounced changes observed with increasing OLE content (Table 3).

**Figure 3:**
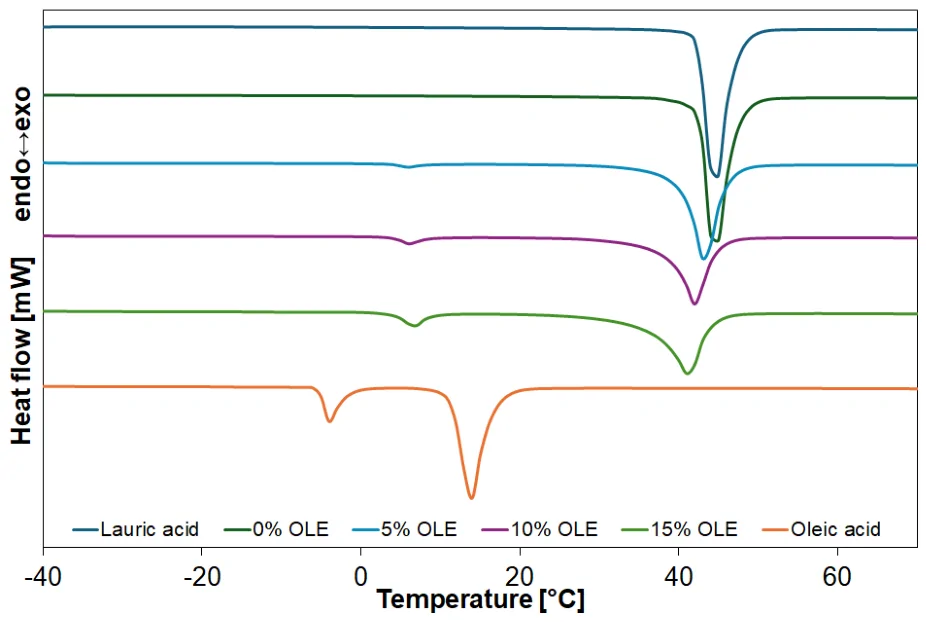
DSC curves of lauric acid, oleic acid, and unloaded NLC microparticles.

As shown in Figure 3 and summarized in Table 4, two endothermic peaks were detected during the melting of pure oleic acid (OLE). The first peak, observed at approximately −5 °C, corresponds to the solid–solid transition from the γ monoclinic polymorph to the α phase, whereas the second peak, at approximately 11 °C, is attributed to the melting of the α phase [27]. In contrast, pure lauric acid exhibited only a single endothermic peak corresponding to its melting at approximately 45 °C. With increasing OLE content, the melting peak of lauric acid gradually decreased, while its melting temperature shifted toward lower values, from 45 °C for pure lauric acid (0% OLE) to approximately 41 °C for 15% OLE. Furthermore, after the incorporation of OLE, the endothermic peak associated with the γ → α solid–solid transition for oleic acid was no longer observed. Instead, only a single endothermic peak corresponding to the melting of the α phase remained, occurring at a lower temperature than that of pure oleic acid. The melting peaks observed for the unloaded nanostructured lipid carriers (NLCs) were considerably smaller than those recorded for pure oleic acid and pure lauric acid (0% OLE). Nevertheless, the melting enthalpy associated with the oleic acid phase gradually increased with increasing OLE content, from 3.4 J/g for the formulation containing 5% OLE to 14.5 J/g for the formulation containing 15% OLE. However, these values remained substantially lower than the melting enthalpy of pure oleic acid.

### 3.2. Tobramycin-Loaded Microparticles

#### 3.2.1. Physicochemical Properties

**Figure 4:**
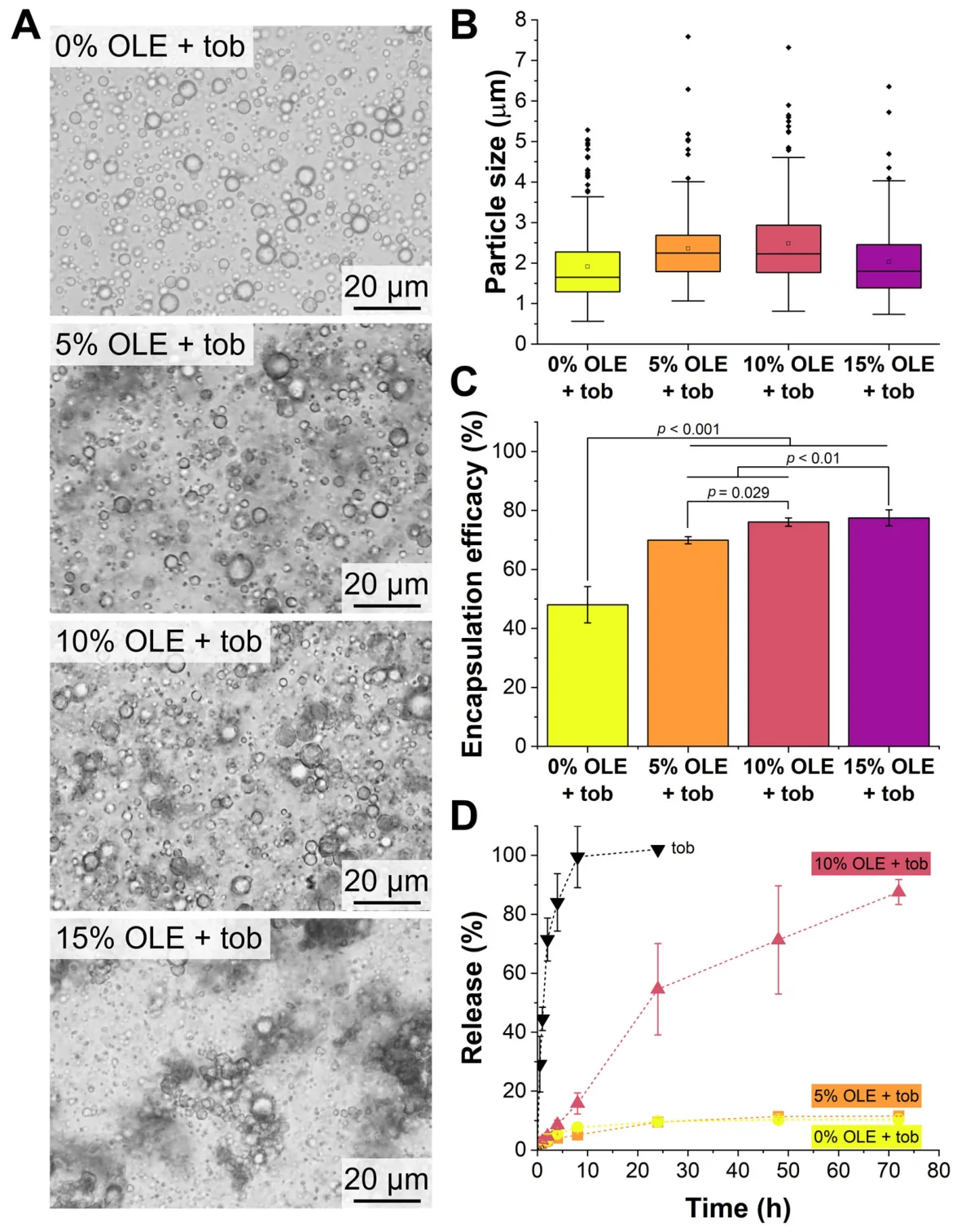
Morphology of nanostructured lipid carrier (NLC) microparticles loaded with tobramycin, evaluated using optical microscopy (**A**), particle size distribution (**B**), encapsulation efficacy of tobramycin determined indirectly using OPA-based amine detection method (**C**), and tobramycin release from nanostructured lipid carrier (NLC) microparticles loaded with tobramycin (**D**).

In terms of microparticle morphology (Figure 4A), no significant influence of tobramycin addition on particle shape, size, or agglomeration was observed. Median particle diameters were in the range of 1.7 µm to 2.2 µm (Figure 4B). Similarly to unloaded samples, a strong tendency for particle agglomeration was observed for 15% OLE + tob microparticles.

In the case of 0% OLE + tob, approximately 48 ± 6% of tobramycin was effectively encapsulated in the microparticles (Figure 4C). The addition of OLE significantly increased the EE of tobramycin to at least 70 ± 1% for 5% OLE + tob and 76 ± 1% for 10% OLE + tob. Further increase in OLE content to 15% did not enhance tobramycin loading (EE equal to 77 ± 3% for 15% OLE + tob). The corresponding DL values were 5.1 ± 0.9%, 7.5 ± 0.4%, 7.9 ± 0.1%, and 8.1 ± 0.3% for 0% OLE + tob, 5% OLE + tob, 10% OLE + tob, and 15% OLE + tob, respectively. Considering lack of EE improvement and strong agglomeration of the microparticles with the highest OLE content, 15% OLE + tob microparticles were excluded from further studies.

One of the most interesting parts of this study was the evaluation of tobramycin release from the microparticles (Figure 4D). For this test, 5 mg of microparticles containing 0.26 ± 0.04 mg, 0.38 ± 0.02 mg, and 0.39 ± 0.01 mg of tobramycin was used for the 0% OLE + tob, 5% OLE + tob, and 10% OLE + tob formulations, respectively. Pure tobramycin (tob) was freely soluble in aqueous Gamble solution, so almost the whole dose was released to the surrounding medium within the first 8 h of incubation. On the contrary, in the case of 0% OLE + tob and 5% OLE + tob, only around 10% of the total tobramycin dose was released from the microparticles within 3 days. In the case of 10% OLE + tob, a gradual release of tobramycin was observed during the whole incubation period—approximately 50% of tobramycin was released within the first 24 h, followed by 20% and 17% of the dose released on the 2nd and 3rd day, respectively. The release profiles were further fitted to different kinetic models, including zero-order, first-order, second-order, and Korsmeyer–Peppas models (Table 5). Among the evaluated models, the Korsmeyer–Peppas model with lag time provided the best fit to the experimental data for all formulations, with R^2^ values ranging from 0.981 to 0.994. In comparison, the zero-order model showed lower fitting accuracy, with R^2^ values between 0.40 and 0.64, while the first- and second-order models showed poor correlation with the experimental data (R^2^ < 0.02).

**Figure 5:**
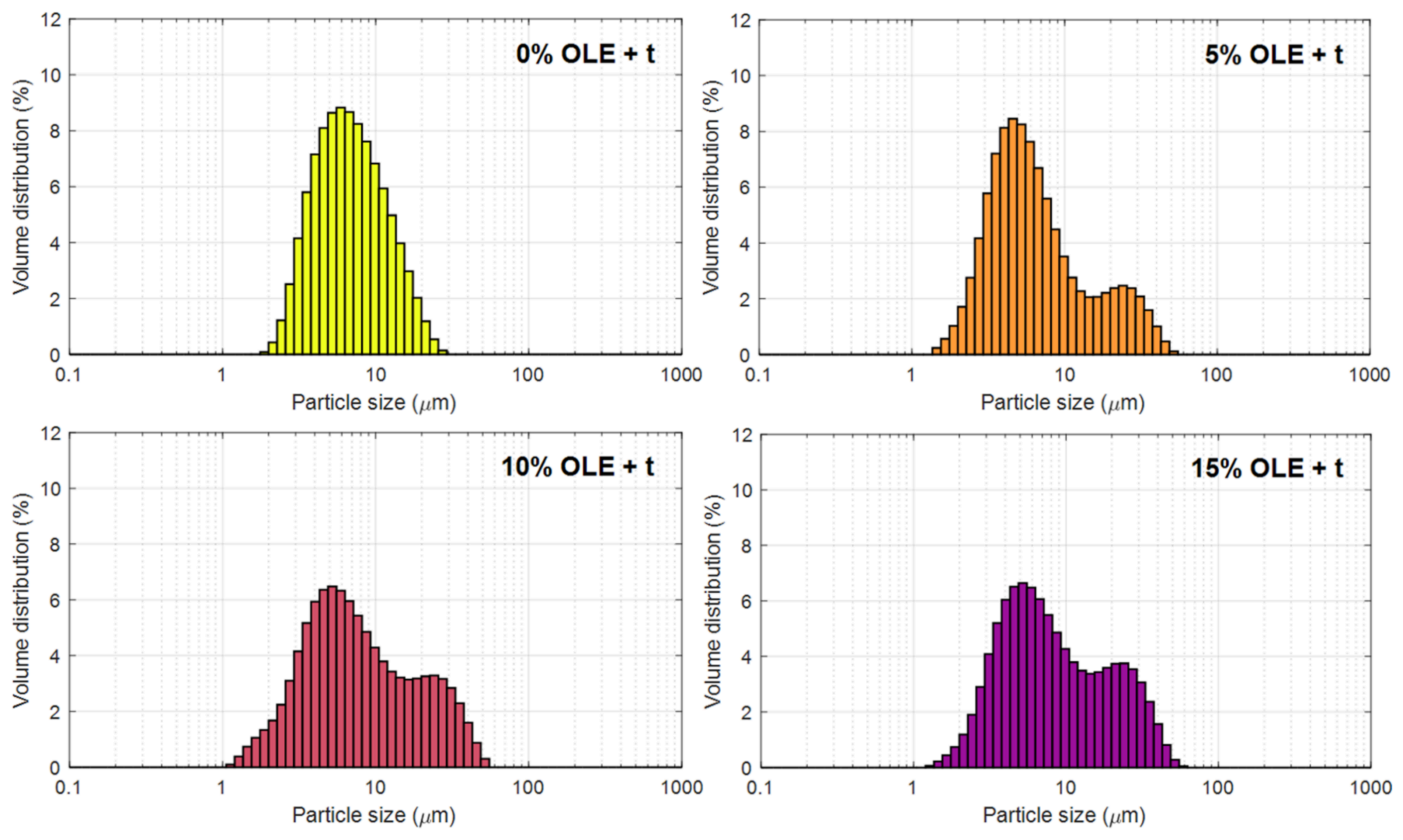
Volume-based particle size distributions of the tobramycin-loaded microparticles determined by laser diffraction.

The particle size distributions of the tobramycin-loaded microparticles are presented in Figure 5. Compared with the unloaded formulations, incorporation of tobramycin resulted in a shift of the particle size distributions toward larger sizes for formulations containing 0%, 5%, and 10% OLE, as reflected by the increased median particle sizes (Dx(50)) of 7.04 μm, 6.09 μm, and 7.47 μm, respectively (Table 6). In contrast, the formulation containing 15% OLE exhibited a comparable particle size distribution to the unloaded microparticles, with a Dx(50) value of 7.91 μm, and no further increase in the contribution of larger particles was observed. The zeta-potential values of the tobramycin-loaded microparticles ranged from approximately −4 to +4 mV (Table 6), indicating a shift toward less negative surface charge compared with the unloaded formulations.

**Figure 6:**
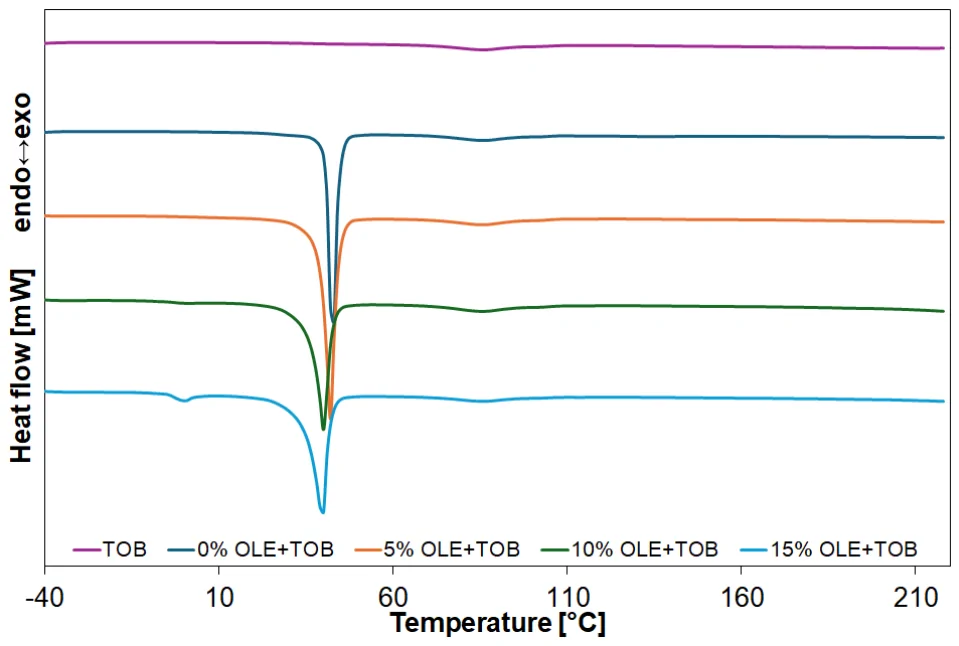
DSC curves of tobramycin and tobramycin-loaded NLC microparticles.

DSC results for NLC microparticles loaded with tobramycin are presented in Figure 6 and in Table 7. As shown in the DSC thermograms, pure tobramycin sulfate exhibited a single thermal effect at approximately 75 °C, which can be attributed to its glass transition, as tobramycin sulfate is an amorphous material [28]. Incorporation of tobramycin sulfate into the nanostructured lipid carriers (NLCs) resulted in a slight decrease in the melting temperature of lauric acid compared with the unloaded NLCs. Furthermore, no melting peak corresponding to oleic acid was observed for the formulation containing 5% OLE. In formulations with higher oleic acid content, the melting temperature of oleic acid decreased from approximately 6–7 °C to around 0 °C. This behavior is most likely associated with hindered crystallization in these multicomponent systems, as previously discussed for the unloaded NLCs. Only minor changes in the glass transition temperature of tobramycin sulfate were observed with varying NLC compositions from 78 °C for 0% OLE to 75 °C for 15% OLE + TOB formulation.

**Figure 7:**
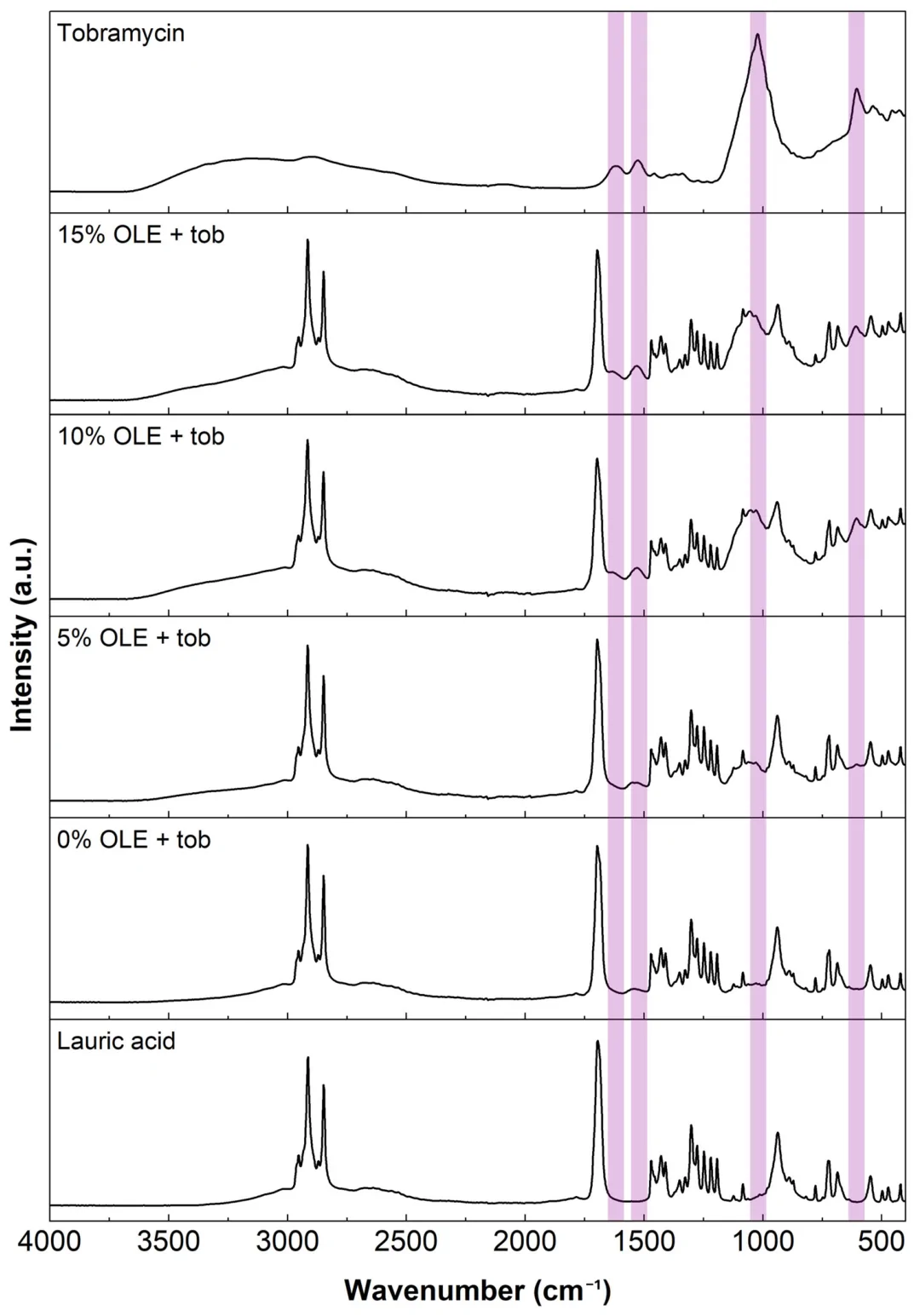
FTIR spectra of nanostructured lipid carrier (NLC) microparticles loaded with tobramycin, together with spectra recorded for pure lauric acid ((**bottom**) panel) and tobramycin ((**top**) panel). Bands characteristic for tobramycin are marked in purple.

FTIR analyses (Figure 7) further confirmed the presence of tobramycin in NLC microparticles. Bands characteristic for fatty acids were identified in all types of microparticles and overlapped with bands visible in the spectra of pure lauric acid. The spectrum of oleic acid was very similar to the spectrum of lauric acid, with the exception of formation of a low-intensity band at around 3008 cm^−1^ originating from C-H groups in alkenes. The presence of CH_2_ groups in fatty acids was confirmed by bands at 2914 cm^−1^ and 2848 cm^−1^ (C-H asymmetrical and symmetrical stretching in CH_2_ groups, respectively), and at 1471 cm^−1^ (CH_2_ deformation); and multiple peaks between 1286 and 1190 cm^−1^ (CH_2_ twisting and wagging), and at 719 cm^−1^ (CH_2_ rocking). A band at 1409 cm^−1^ was assigned to CH_2_ deformation at C2, i.e., carbon atom next to COOH group. A single CH_3_ group could be identified based on bands at 2954 cm^−1^ and 2871 cm^−1^ (C-H asymmetrical and symmetrical stretching in CH_3_ groups, respectively), 1350 cm^−1^ (CH_3_ deformation), and 1122 cm^−1^ (CH_3_ rocking). Carboxylic group (-COOH) resulted in a strong band at 1697 cm^−1^, originating from C=O stretching in hydrogen-bonded fatty acid dimers; a band at 1429 cm^−1^ (C-O stretching); and several peaks from -OH group (wide band in the range of 3500–2660 cm^−1^ from O-H stretching and a band at 930 cm^−1^ from OH out-of-plane bending) [29,30,31]. Tobramycin can be identified using bands at around 1620 cm^−1^ and 1525 cm^−1^, originating from N-H bending; a strong band at around 1025 cm^−1^ related to C-N/C-O stretching vibrations; and a band at 605 cm^−1^, attributed to asymmetrical bending of a sulphate group [32,33,34]. The intensity of bands originating from tobramycin increased proportional to the increase in tobramycin content in the microparticles, confirming successful loading of the drug into the microparticles. No significant differences were observed between the samples containing 10% and 15% OLE.

Final evaluation of physicochemical properties of the samples was done in terms of their aerodynamic properties (Table 8). Theoretical aerodynamic diameters calculated based on the diameters of the particles measured using optical microscopy images, bulk and tapped densities, were in the range of 0.8 µm (for 0% OLE + tob) to 1.1 µm (10% OLE + tob). Interestingly, the addition of oleic acid to the microparticles improved their flowability, as evidenced by decreased CI and HR. Flow character changed from fair/passable for 0% OLE + tob to good in the case of 5% OLE + tob and 10% OLE + tob.

#### 3.2.2. Antibacterial Properties

Prior the antibacterial activity assessment of the drug-loaded microparticles, the standard tests of susceptibility of the chosen strains to tobramycin were carried out using standard E-Test strips. The evaluated values were 0.19, 2, 1, and 12 µg/mL for *S. aureus*, *P. aeruginosa*, *E. coli*, and *K. pneumoniae*, respectively.

**Figure 8:**
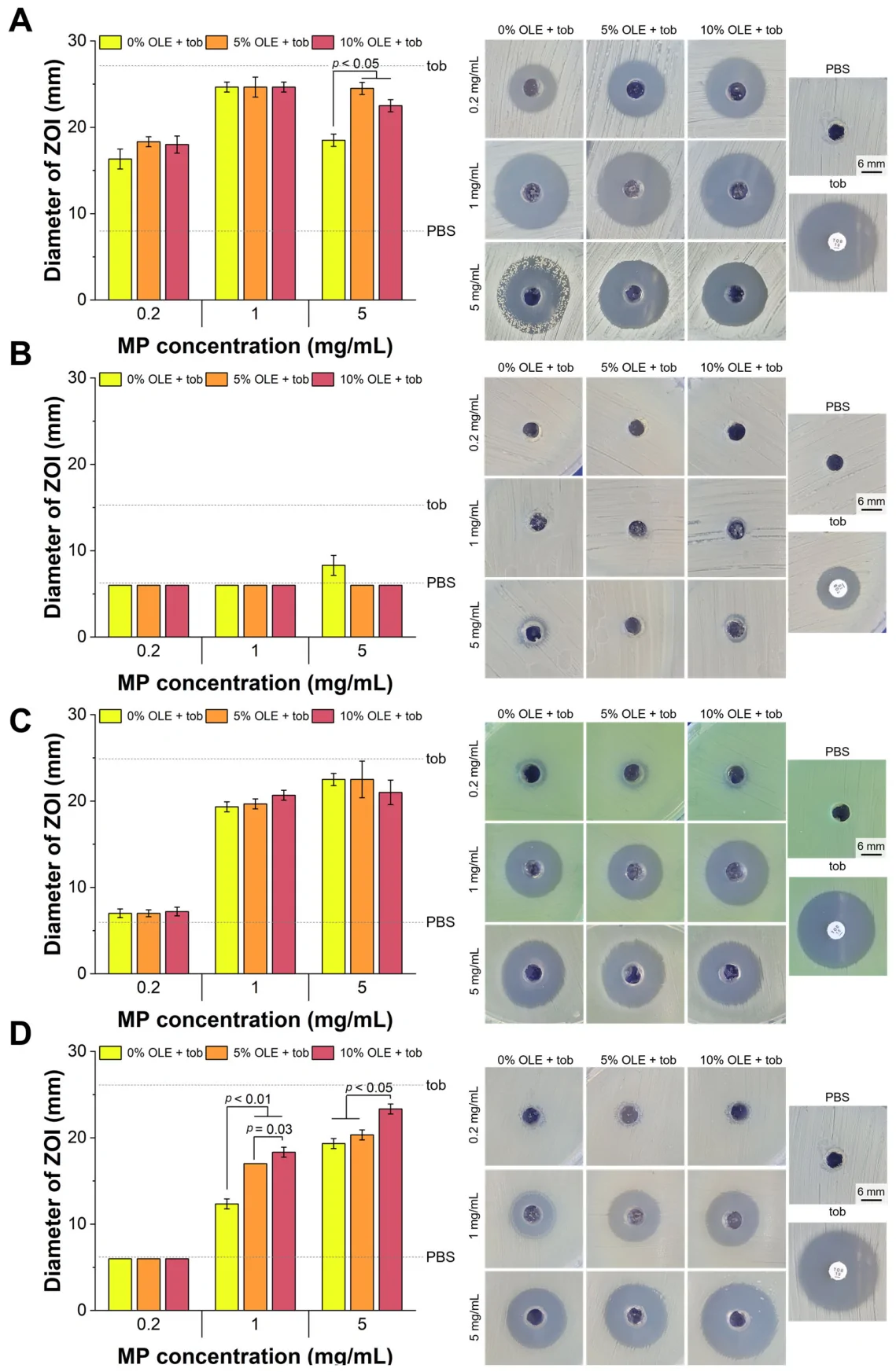
Antibacterial efficacy of nanostructured lipid carrier (NLC) microparticles loaded with tobramycin, determined via Kirby–Bauer agar diffusion assay, against reference strains of *S. aureus* ATCC 25923 (**A**), *K. pneumoniae* ATCC 700603 (**B**), *P. aeruginosa* ATCC 27853 (**C**), and *E. coli* ATCC 25922 (**D**).

The strongest antibacterial activity of the microparticles was observed against *S. aureus* (Figure 8A) and *P. aeruginosa* (Figure 8C) bacterial strains. In the first case, even the lowest dose of the microparticles, i.e., 0.2 mg/mL, was effectively hampering bacterial growth, while in the second, 1 mg/mL of the microparticles was necessary for bacterial growth inhibition. A moderate antibacterial efficacy of the microparticles was observed against *E. coli* strain (Figure 8D); however, in this case, a strong dose-dependent response was present, both in terms of the dose of microparticles (higher efficacy at a concentration of 5 mg/mL in comparison to 1 mg/mL) and in terms of the tobramycin loading (stronger inhibition in the case of 5% OLE + tob and 10% OLE + tob, in contrast to 0% OLE + tob). *K. pneumoniae* (Figure 8B) was the least prone to tobramycin-loaded microparticles; almost no growth inhibition was observed even at the highest concentration of the microparticles. However, it was more likely related to the efficacy of tobramycin alone, as, in this case, tobramycin itself (standard disc) formed the smallest growth inhibition zone (15 mm in diameter, compared to at least 25 mm in all other strains).

**Figure 9:**
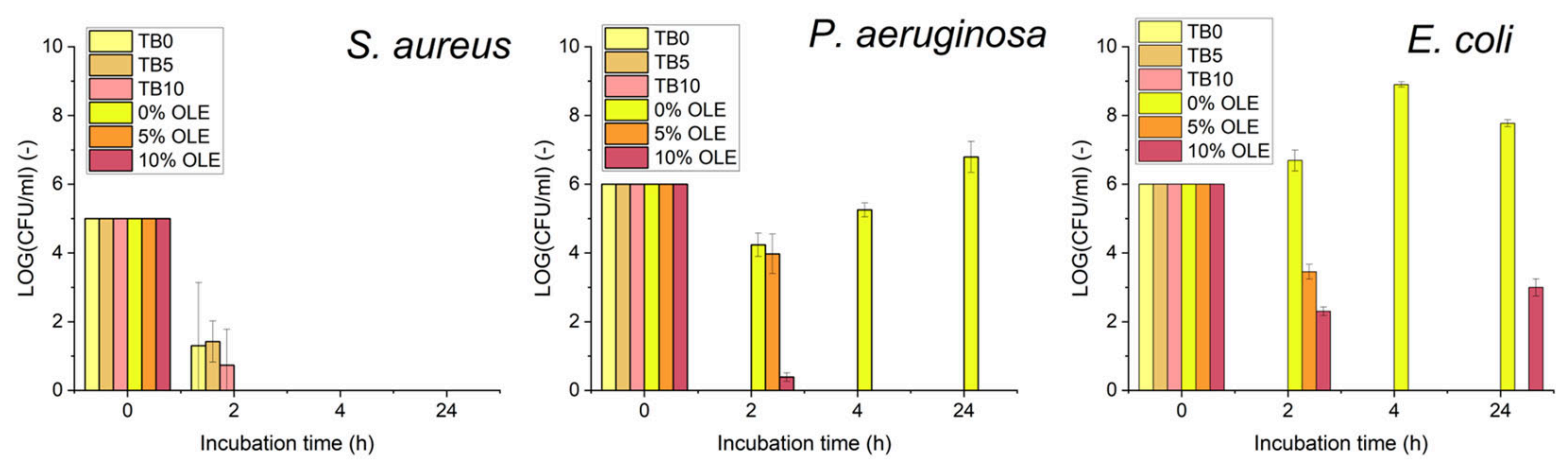
Time-kill assay of nanostructured lipid carrier (NLC) microparticles loaded with tobramycin vs. free tobramycin dose respective to the loading of the NLC determined for *S. aureus* ATCC 25923, *P. aeruginosa* ATCC 27853, and *E. coli* ATCC 25922.

Following the Kirby–Bauer initial antibacterial properties’ evaluation, a time-kill assay was performed for the concentration of 1 mg/mL of the particles and respective tobramycin doses assessed from the DL values presented in Section 3.2.1, with the exclusion of *K. pneumoniae*, for which no antibacterial efficacy was observed (Figure 9).

Against *S. aureus*, all tested formulations exhibited rapid bactericidal activity. Free tobramycin (TB0, TB5, and TB10) reduced the bacterial count by more than 3 log_10_ within 2 h, while all tobramycin-loaded microparticle formulations (0% OLE, 5% OLE, and 10% OLE) reduced the bacterial count below the limit of detection after 2 h. According to CLSI criteria, the absence of detectable colonies corresponds to bacterial counts below the detection limit rather than absolute sterility, and therefore represents a bactericidal effect (>4 log_10_ reduction) [35]. No bacterial regrowth was observed after 4 or 24 h for any tested formulation.

For *P. aeruginosa*, free tobramycin produced complete bactericidal activity within 2 h. In contrast, the microparticle formulations showed oleic acid-dependent activity. After 2 h, the 0% OLE and 5% OLE formulations reduced bacterial counts but did not reach the bactericidal threshold, whereas the 10% OLE formulation achieved a >3 log_10_ reduction. After 4 h, complete bactericidal activity was observed for both the 5% OLE and 10% OLE formulations, while the 0% OLE formulation remained ineffective. After 24 h, bacterial regrowth was observed in the 0% OLE group, whereas no viable bacteria were detected in the 5% OLE and 10% OLE groups.

A similar trend was observed for *E. coli*. Free tobramycin completely eliminated viable bacteria within 2 h. Among the microparticle formulations, the 0% OLE formulation showed no antibacterial activity, and bacterial growth continued throughout the experiment. In contrast, both the 5% OLE and 10% OLE formulations produced a bactericidal effect after 2 h. Complete eradication of viable bacteria was observed after 4 h for the 5% OLE formulation and after 24 h for both oleic acid-containing formulations, whereas the 0% OLE formulation failed to inhibit bacterial growth.

Overall, incorporation of oleic acid markedly enhanced the antibacterial efficacy of the lipid microparticles against Gram-negative bacteria. While all formulations were highly effective against *S. aureus*, only microparticles containing 5% or 10% oleic acid exhibited sustained bactericidal activity against *P. aeruginosa* and *E. coli*, with the 10% OLE formulation providing the fastest antibacterial response.

### 3.3. In Vitro Cytotoxicity

**Figure 10:**
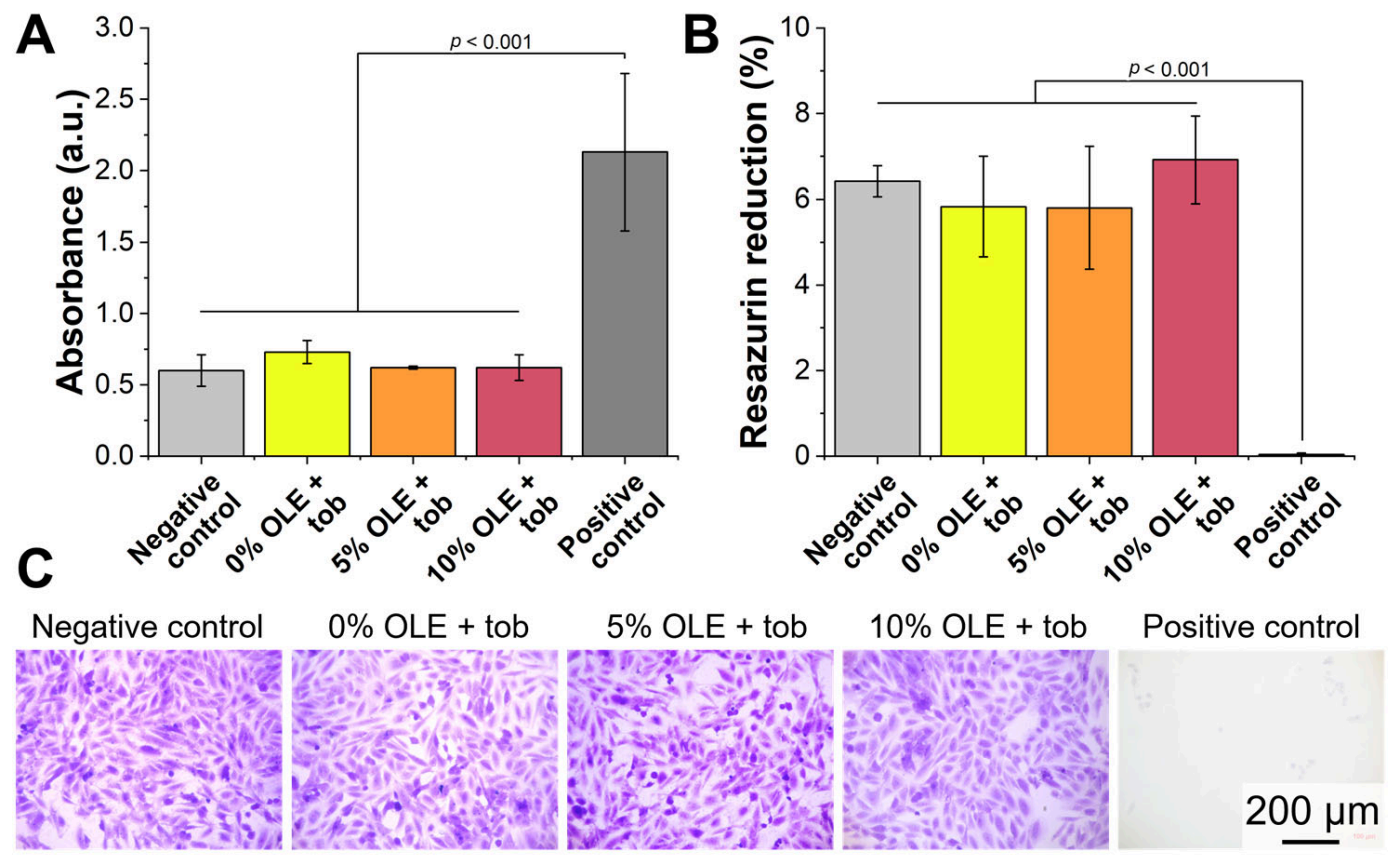
Cytotoxicity of nanostructured lipid carrier (NLC) microparticles tested on BEAS-2B lung epithelial cells: release of LDH (**A**), metabolic activity of cells assessed by resazurin reduction test (**B**), and cell morphology after crystal violet staining (**C**). Negative control, cells cultured in cell culture medium; positive control, cells treated with Triton-X 100.

Similarly to unloaded microparticles, the microparticles loaded with tobramycin were cytocompatible with BEAS-2B non-malignant lung epithelial cells, as evidenced by LDH release (Figure 10A) and metabolic activity (Figure 10B) assays. No statistically significant differences were found between tested microparticles and negative control. The morphology of the cells visualized using crystal violet staining (Figure 10C) revealed that the apparent number of cells, and their shape and spread were similar to that of the untreated control.

## 4. Discussion

This study evaluated the feasibility of fabricating fatty acid-based NLC microparticles for pulmonary delivery of tobramycin. Particular attention was given to achieving a particle size within the respirable range, enhancing drug loading through incorporation of a liquid lipid, and preserving antibacterial activity. The findings obtained are discussed in the context of these objectives.

The hot emulsification method used in this study was suitable for manufacturing of the nanostructured microparticles based on a mixture of lauric and oleic acids. Tobramycin is highly stable toward hydrolysis at neutral pH, with a reported t_90_, defined as the time required for 10% degradation, of approximately 70 h at 80 °C in phosphate buffer (pH 7) [36]. Therefore, heating to 65 °C during the fabrication process is very unlikely to compromise its structural integrity or biological activity. No organic solvents were necessary for solubilization of the organic phase, as is necessary in other solvent-based emulsification methods; thus, the obtained microparticles were for sure not contaminated with any remnants of possibly toxic solvents. What is more, the hot oil-in-water emulsification method can be easily scaled up using microfluidic devices, thus facilitating clinical translation of the developed microparticles.

DSC analysis confirmed the composition of the NLCs and demonstrated the effect of formulation composition on their phase transitions. The reduced melting enthalpy observed for both fatty acids can be attributed to hindered crystallization in the binary mixtures. The presence of the second component disrupts the crystal lattice, resulting in less efficient molecular packing and the formation of amorphous or poorly ordered regions. In addition, the double bond in oleic acid introduces a permanent bend in the hydrocarbon chain that hinders efficient packing with neighboring saturated lauric acid molecules and inhibits crystal growth. Consequently, the degree of crystallinity and the heat required for melting are reduced. Such behavior is characteristic for binary fatty acid mixtures and has also been reported for lauric acid/oleic acid systems and other lauric acid-based mixtures [37,38]. Incorporation of tobramycin sulfate also affected the phase behavior of the NLCs. In general, it decreased the phase transition temperatures and enthalpies of both fatty acids. Moreover, minor changes in the glass transition temperature of tobramycin sulfate were observed with varying NLC composition. The formulation containing 0% OLE exhibited the highest glass transition temperature (78 °C), which can be explained by the restriction of molecular mobility of the amorphous tobramycin sulfate by the surrounding crystalline lauric acid domains. As the OLE content increased, the crystallization ability of lauric acid decreased, leading to a less rigid lipid matrix and, consequently, to a gradual decrease in the glass transition temperature of tobramycin sulfate to 75 °C for 15% OLE + TOB.

The morphology of the microparticles did not differ significantly regardless of the amount of liquid lipid (OLE) or the addition of tobramycin. The only exception was the formulation containing 15% OLE, for which strong aggregation of the microparticles was observed in both unloaded and tobramycin-loaded samples. This behavior may be attributed to the presence of an excessive amount of liquid lipid, which could not be effectively accommodated within the solid lipid matrix. As oleic acid remains liquid at room temperature, its partial expulsion from the lipid structure may have promoted particle–particle interactions and subsequent aggregation. Similar instability has been reported for oleic acid-based lipid nanocarriers, where the ratio between liquid lipid, solid lipid, and stabilizing components strongly influences structural organization and colloidal stability, with suboptimal compositions leading to flocculation, coalescence, or aggregation phenomena [39,40].

These observations were further supported by particle size analysis of the unloaded microparticles. Formulations containing 0%, 5%, and 10% OLE exhibited comparable median particle sizes, remaining within the range generally considered suitable for pulmonary delivery. In contrast, the 15% OLE formulation showed a pronounced increase in median particle size (Dx(50) = 12.7 μm) and a broader size distribution compared with formulations containing lower OLE concentrations. The increased contribution of larger particles to the volume-weighted distribution provides further evidence of particle aggregation. This phenomenon is undesirable for pulmonary delivery applications, as particle size strongly influences aerosolization behavior and deposition within the respiratory tract [5,7,10]. Therefore, the addition of 15% OLE appeared to exceed the optimal liquid lipid content for this formulation, resulting in unfavorable particle characteristics and leading to its exclusion from further investigations.

In contrast, the zeta-potential values of unloaded microparticles remained relatively consistent across all OLE concentrations, suggesting that incorporation of increasing amounts of OLE mainly affected the internal lipid organization rather than the surface charge properties of the particles.

Following tobramycin incorporation, an increase in median particle size was observed for formulations containing 0%, 5%, and 10% OLE, despite no evident differences being identified during optical microscopy analysis. This apparent discrepancy can be attributed to differences between the analytical approaches. Laser diffraction provides a volume-weighted particle size distribution and is therefore highly sensitive to a small fraction of larger particles or loosely associated aggregates, as these can significantly affect the calculated volume distribution. In contrast, optical microscopy combined with manual ImageJ analysis provides mainly number-based information and may not adequately represent minor populations of larger particles contributing to the overall volume distribution.

Interestingly, tobramycin loading resulted in a shift of the zeta-potential values toward near-neutral values (approximately −4 to +4 mV). This change may be attributed to interactions between positively charged amino groups of tobramycin and negatively charged lipid components at the particle surface. The gradual decrease in ζ-potential with increasing oleic acid content may indicate differences in the localization or interaction of tobramycin within the lipid matrix and at the particle interface. Near-neutral surface charge may be advantageous for pulmonary delivery, as highly charged particles can exhibit stronger interactions with airway mucus components, potentially limiting mucus penetration. In contrast, particles with reduced surface charge may demonstrate decreased mucoadhesion and improved mobility through the mucus layer, which may facilitate deeper deposition within the respiratory tract [7,8].

The aerodynamic evaluation of the NLC microparticles confirmed that the incorporation of oleic acid had a beneficial effect on their flowability. The calculated theoretical aerodynamic diameters remained consistently in the respirable range (0.8–1.1 µm), indicating suitability for pulmonary delivery across all formulations. Importantly, increasing OLE content did not adversely affect particle dispersion characteristics; rather, it contributed to improved powder-handling properties. This improvement was reflected in the bulk flow parameters, where both CI and HR decreased with increasing OLE concentration. The formulation without oleic acid exhibited only fair flowability (CI ca. 21.5%, HR ca. 1.21), whereas the addition of OLE reduced interparticle cohesion, yielding values indicative of good flow (CI ca. 12%, HR ca. 1.12). For example, a Montelukast-loaded NLC dry powder formulation with a solid-to-liquid lipid ratio of 7:3 exhibited a CI of 21% and an HR of 1.28 [41]. Overall, these changes indicate a transition toward a less cohesive and more free-flowing system, which is advantageous for further processing and inhalation performance [42]. However, it should be noted that the present aerodynamic assessment reflects only the flowability characteristics of the microparticles and does not represent a complete evaluation of aerosolization performance. Comprehensive assessment of dry powder inhaler formulations requires cascade impactor analysis to determine parameters such as emitted dose and fine particle fraction. Due to their lipid composition and associated cohesive interactions, lipid microparticles may require incorporation of suitable carrier excipients, such as lactose, to improve powder dispersibility and aerosolization efficiency. The addition of such excipients has been widely explored in inhalable dry powder formulations, where carrier particles reduce interparticle cohesion and promote efficient dispersion during inhalation [43]. Therefore, future optimization of the developed formulation should include blending with an appropriate inhalation-grade excipient, followed by cascade impactor evaluation to confirm its suitability for pulmonary delivery.

Our initial assumption, that the addition of liquid lipid to the matrix of the microparticles would increase the encapsulation efficacy of tobramycin, was confirmed. Indeed, EE increased significantly from approximately 50% in 0% OLE + tob to approximately 75% in 10% OLE + tob. Further increase in OLE content did not enhance tobramycin loading. Considering this, and the fact that a 15% addition of OLE lead to agglomeration of the microparticles, 15% OLE + tob was excluded from the study.

Interestingly, even the microparticles composed solely of solid lipid (0% OLE + tob) exhibited relatively high tobramycin encapsulation despite the strongly hydrophilic nature of the drug (water solubility ca. 50 mg/mL). Encapsulation of hydrophilic compounds in lipid-based nanoparticles is generally challenging due to their preferential partitioning into the aqueous phase during preparation. For comparison, the encapsulation efficiency of trypsin-loaded SLNs has recently been reported to be below 50% for an optimized formulation based on cetyl palmitate and Pluronic F68 [41]. To explain this phenomenon, a chemical structure of both fatty acids and tobramycin (in a form of sulfate salt), and possible interactions between these two compounds should be analyzed. In fact, each fatty acid molecule contains one carboxylic group that can easily react with amino cations present in tobramycin. Pignatello et al. [33] proved that once a pentasulfate salt of tobramycin is mixed with lipoamino acids (α-amino acids with long acyl side chains), amino cations in tobramycin form new complexes with carboxylic anions in lipoamino acids. This phenomenon of forming ionic associates between two functional groups is known as “hydrophobic ion pairing” [44,45] and can be applied to increase the lipophilicity of different hydrophilic compounds. Fatty acids, such as oleic or stearic acids, have already been studied in this context, as once the lipophilic acid residues complex amine groups in normally hydrophilic moieties (i.e., gentamycin and tobramycin), they reduce the water solubility of the moiety and enable its encapsulation in organic matrix (lipid or polymeric carriers). We hypothesize that the formation of ionic complexes between carboxylic groups in fatty acids and amine groups in tobramycin contributed to such a high EE in 0% OLE + tob, which was further increased by the formation of the nanostructured solid–liquid matrix of the microparticles containing oleic acid.

Strong interactions between tobramycin and the lipid components may also contribute to the limited release observed for 0% OLE + tob and 5% OLE + tob. In aqueous environment of Gamble solution, tobramycin was retained within the microparticles. We hypothesize that, in the case of 10% OLE + tob, the complexes between tobramycin and fatty acids were also formed; however, due to a relatively high content of liquid lipid and the formation of an imperfect solid–liquid matrix, the microstructure of the microparticles was not homogenous and was more prone to infiltration with water, leading to disruption of the microparticles and gradual release of tobramycin.

The kinetic analysis further supported the complex nature of tobramycin release from the developed microparticles. The release behavior was best described by the Korsmeyer–Peppas model with lag time, indicating that drug liberation was not governed by a simple concentration-dependent process. This model is commonly applied to controlled-release systems in which multiple factors, including diffusion through the carrier structure and formulation-related changes during hydration, may contribute to drug release [46,47]. The presence of a lag time suggests that initial interaction between the aqueous environment and the lipid matrix, including water penetration and structural rearrangement of the microparticles, may precede measurable drug diffusion.

It should also be noted that the duration of the in vitro release experiment does not directly correspond to the pulmonary residence time of the formulation in vivo. Following inhalation, particle retention is governed by multiple physiological processes, including dissolution, mucociliary clearance, alveolar macrophage uptake, epithelial transport, and regional deposition, all of which are strongly formulation-dependent. Consequently, pulmonary retention may vary from several hours to several days depending on the physicochemical properties of the carrier [48,49]. Therefore, the prolonged release observed over 72 h should be interpreted as evidence of the sustained-release capability of the developed formulation under controlled in vitro conditions rather than as a direct prediction of its in vivo behavior. Future pharmacokinetic studies will be required to establish the actual pulmonary residence time and drug-release profile following inhalation.

The microbiological assessment further confirmed the functional release of tobramycin from the developed microparticles. Pronounced inhibition of *S. aureus* and *P. aeruginosa* demonstrated that sufficient amounts of the encapsulated antibiotic were released to exert a biological effect, even at relatively low microparticle concentrations. The inhibition of *P. aeruginosa* is particularly relevant for inhaled tobramycin delivery, as this pathogen is a major cause of chronic respiratory infections and can form persistent biofilms that reduce antibiotic susceptibility, making the maintenance of effective local antibiotic concentrations essential [20,21]. These findings indicate that the sustained-release profile was sufficient to achieve antibacterial activity against clinically relevant pathogens.

It is worth noting that the Kirby–Bauer test did not reflect the release from the microparticles. The inhibition zones were similar for each bacterial strain between the samples with different OLE ratios. The time-kill assay, however, showed data consistent with the release—microparticles obtained partly from OLE released drug faster and were much more efficient in eradicating Gram-negative bacteria. This observation is consistent with the observation that oleic acid alone is not an efficient antibacterial agent against *P. aeruginosa* or *E. coli*, but may enhance efficacy as the carrier in, e.g., liposomal formulations [50,51]. Interestingly, microparticles were slightly more effective against Gram-positive bacteria than tobramycin alone for all formulations. This phenomenon may come from the natural ability of lauric acid to affect the viability of *Staphylococcal* strains [52,53]. The synergistic activity of oleic acid and tobramycin has not been studied, to the best of our knowledge, but it was reported for tobramycin with another unsaturated fatty acid, i.e., linolenic acid [54,55].

In addition to the released drug, the lipid matrix itself may also have contributed to the observed antibacterial effect. Lauric acid is well known for its intrinsic antimicrobial activity, particularly against *S. aureus* [56,57], and previous studies have shown that fatty acids can disrupt bacterial membranes, increasing membrane permeability and enhancing bacterial susceptibility to antibiotics [55,58]. Such membrane-disruptive effects may facilitate the interaction of tobramycin with bacterial cells by increasing accessibility to intracellular targets, thereby contributing to the overall antibacterial activity of lipid-based antibiotic carriers [23,59]. Therefore, the pronounced inhibition observed for *S. aureus* may reflect not only the activity of released tobramycin but also a complementary contribution from lauric acid. Similar synergistic interactions have been reported for other fatty acids, such as myristoleic acid, which has been shown to potentiate the antibacterial activity of tobramycin against *S. aureus* [55,58]. Consequently, optimization of the lipid composition may represent a promising strategy for further enhancing the antibacterial performance of lipid-based microparticles.

In contrast, *E. coli* exhibited a more pronounced dose-dependent response, indicating that higher microparticle concentrations were required to achieve effective inhibition. This lower susceptibility compared with *S. aureus* may be related to the additional outer membrane present in Gram-negative bacteria that contains lipopolysaccharides and represents an important permeability barrier against antimicrobial compounds. Differences in outer-membrane composition and organization between Gram-negative species may further influence their susceptibility to lipid-based antimicrobial systems [60,61,62]. This observation is consistent with the lower susceptibility of Gram-negative bacteria to both fatty acids and aminoglycosides, due to differences in cell envelope architecture and antimicrobial defense mechanisms. Likewise, the negligible activity observed against *K. pneumoniae* is likely attributable to its well-recognized intrinsic resistance mechanisms, rather than insufficient drug release from the microparticles.

Furthermore, the clinical relevance of the developed formulation depends on achieving an appropriate therapeutic dose of tobramycin. The approved dry powder inhaler formulation TOBI Podhaler^®^ delivers 112 mg of tobramycin per administration, corresponding to four capsules containing 28 mg of drug each [63]. In the present study, the optimized formulation achieved an encapsulation efficiency of approximately 80%, resulting in a tobramycin content of approximately 82 mg per gram of freeze-dried microparticles. Consequently, achieving a comparable nominal dose would, at this stage, require a higher amount of powder. However, direct comparison with currently approved formulations should be interpreted with caution, as therapeutic efficacy depends not only on the nominal drug dose but also on multiple formulation- and device-related factors, including aerosolization efficiency, pulmonary deposition, and drug-release behavior [64].

The cytotoxicity evaluation confirmed that both unloaded and tobramycin-loaded microparticles were well tolerated by BEAS-2B lung epithelial cells, with no significant effects on membrane integrity or metabolic activity. The assessment was performed using a relatively high particle concentration of 100 µg/mL, selected as a stringent testing condition to evaluate potential cytotoxic effects under an exaggerated exposure scenario. Previous studies on inhalable microparticle systems have shown that elevated particle concentrations may negatively affect cell viability due to increased particle deposition and physical interactions with the cell layer, rather than intrinsic toxicity of the carrier material itself [65,66]. Therefore, the absence of cytotoxic effects at the applied concentration further supports the favorable biocompatibility of the developed formulations. Importantly, the lack of differences between formulations with varying OLE content suggests that incorporation of the liquid lipid did not adversely affect cytocompatibility. The preserved cell morphology further supports the absence of detectable cellular damage. These findings are consistent with previous reports of good biocompatibility of lipid-based carriers and support the suitability of the developed microparticles for pulmonary delivery applications.

Overall, the obtained results demonstrate that fatty acid-based NLC microparticles can be successfully engineered to achieve respirable aerodynamic properties, effective tobramycin encapsulation, and preserved antimicrobial activity, while maintaining acceptable cytocompatibility. The system appears particularly sensitive to the balance between solid and liquid lipid fractions that governs both structural stability and drug-release behavior. Nevertheless, the current study was limited to in vitro evaluation, and further investigations are required to assess the in vivo biodistribution, pharmacokinetics, pharmacodynamics, and therapeutic efficacy of the developed formulation following pulmonary administration. Further studies should therefore focus on more physiologically relevant pulmonary models, including Calu-3 epithelial monolayers, to better assess barrier transport and mucus interactions, as well as advanced co-culture systems incorporating airway cells and bacterial biofilms to more closely mimic infection conditions. In addition, in vivo inhalation studies and deeper investigation of the drug–lipid interaction mechanisms would be valuable to fully establish the translational potential of the developed system. Future work should also include the detailed optimization of the inhalable formulation, including excipient selection and refinement of the powder properties, to further improve aerosol performance and clinical applicability.

## 5. Conclusions

LRTIs remain a major global health burden and continue to require improved therapeutic strategies, particularly those that enable a more effective local delivery of antibiotics to the lungs. To address this need, we developed fatty acid-based NLC microparticles, using a hot emulsification method, as active carriers with potential for the pulmonary delivery of tobramycin.

The systems obtained exhibited aerodynamic diameters within the respirable range, indicating their potential for further investigation in inhalation applications. Incorporation of 5–10% (*w*/*w*) oleic acid improved tobramycin-encapsulation efficiency up to approximately 75%, with no further benefit observed at 15% (*w*/*w*) concentration, which additionally resulted in strong particle agglomeration and unfavorable size characteristics. The lipid composition strongly influenced the physicochemical properties and drug-release behavior of the microparticles. The presence of oleic acid contributed to the formation of a less crystalline lipid matrix, which enhanced drug loading and promoted more effective tobramycin release under simulated pulmonary conditions. In contrast, formulations with lower OLE content exhibited stronger drug retention, likely due to tighter interactions between tobramycin and the fatty acid matrix.

All formulations demonstrated cytocompatibility with BEAS-2B lung epithelial cells and preserved antibacterial activity against clinically relevant respiratory pathogens. The observed antimicrobial effects resulted from successful tobramycin delivery, together with the inherent antibacterial contribution of the fatty acid components, particularly against Gram-positive bacteria.

Overall, these findings support the potential of fatty acid-based nanostructured lipid microparticles as carriers of tobramycin in pulmonary drug delivery, combining efficient drug encapsulation, favorable particle characteristics, sustained-release capability, and biological activity. Further studies, including optimization of powder formulation, aerosol performance evaluation using cascade impactor analysis, and validation in more physiologically relevant pulmonary models, are required to establish their suitability for use in a final DPI formulation and further assess their translational potential.

## Figures and Tables

**Table 1:** Composition of unloaded microparticles.

Sample	Lauric Acid [% *w*/*w*]	Oleic Acid [% *w*/*w*]
0% OLE	100	0
5% OLE	95	5
10% OLE	90	10
15% OLE	85	15

**Table 2:** Composition of tobramycin-loaded microparticles.

Sample	Lauric Acid [% *w*/*w*]	Oleic Acid [% *w*/*w*]	Tobramycin [% *w*/*w*]
0% OLE + tob	90.0	0.0	10
5% OLE + tob	85.5	4.5	10
10% OLE + tob	81.0	9.0	10
15% OLE + tob	76.5	13.5	10

**Table 3:** Median particle size (Dx(50)), span, and zeta (ζ) potential of the unloaded microparticles.

Sample	Dx(50) [μm]	Span	ζ-Potential [mV]
0% OLE	4.05	2.47	−19.1 ± 4.2
5% OLE	3.12	1.57	−14.2 ± 3.5
10% OLE	4.66	1.82	−17.2 ± 3.4
15% OLE	12.70	3.95	−16.6 ± 4.3

**Table 4:** Temperature and heat of melting of lauric acid, oleic acid, and unloaded NLC microparticles.

Samples	Melting Temperature [°C]			Heat of Melting [J/g]	Heat of Melting (Normalized to the Acid Content in the System) [J/g]
	T_onset_	T_peak_	T_endset_		
Lauric acid	42	45	47	182.6	182.6
0% OLE	43	44	47	182.1	182.1
5% OLE	4	6	8	3.4	68.0
	40	43	45	152.1	160.1
10% OLE	4	6	8	7.7	77.0
	38	42	44	119.5	132.8
15% OLE	4	7	9	14.5	96.7
	36	41	44	129.7	152.6
Oleic acid	−5	−4	−2	27.8	27.8
	11	14	16	136.2	136.2

**Table 5:** Coefficients of determination (R^2^) obtained from fitting the release profiles to different kinetic models.

Sample	0-Order Model	1-Order Model	2-Order Model	Korsmeyer–Peppas with Lag
	R^2^	R^2^	R^2^	R^2^
0% OLE + tob	0.64 ± 0.08	0.010 ± 0.001	0.004 ± 0.001	0.981 ± 0.004
5% OLE + tob	0.4 ± 0.1	0.010 ± 0.001	0.004 ± 0.001	0.985 ± 0.004
10% OLE + tob	0.52 ± 0.05	0.014 ± 0.001	0.004 ± 0.001	0.994 ± 0.002

**Table 6:** Particle size characteristics and zeta (ζ) potential of the tobramycin-loaded microparticles.

Sample	Dx(50) [μm]	Span	ζ-Potential [mV]
0% OLE	7.04	1.59	3.5 ± 4.6
5% OLE	6.09	3.48	0.3 ± 3.1
10% OLE	7.47	3.42	−2.5 ± 3.0
15% OLE	7.91	3.25	−4.4 ± 3.8

**Table 7:** Temperature and heat of melting of tobramycin and tobramycin-loaded NLC microparticles.

Samples	Melting Temperature [°C]			Glass Temperature [°C]	Heat of Melting [J/g]	Heat of Melting (Normalized to the Acid Content in the System) [J/g]
	T_onset_	T_peak_	T_endset_			
Tobramycin	67	86	100	75	19.6	19.6
0% OLE + TOB	41	43	44	78	87.3	97.0
5% OLE + TOB	-	-	-	77	0	0
	39	42	44		122.3	143.1
10% OLE + TOB	−5	0	6	77	1.1	12.2
	36	40	42		95.2	117.5
15% OLE + TOB	−4	0	3	75	4.4	32.6
	35	40	42		102.1	133.5

**Table 8:** Flow characteristics of nanostructured lipid carrier (NLC) microparticles loaded with tobramycin.

Sample	d_g_ [µm]	ρ [g/cm^3^]		D_aero_ [µm]	CI [%]	HR [-]	Flow Character
		Bulk	Tapped				
0% OLE + tob	1.9 ± 0.9	0.15 ± 0.01	0.18 ± 0.01	0.8 ± 0.4	21.5 ± 5.7	1.21 ± 0.06	Fair/passable
5% OLE + tob	2.4 ± 0.9	0.16 ± 0.05	0.18 ± 0.06	1.0 ± 0.4	12.5 ± 6.8	1.12 ± 0.07	Good
10% OLE + tob	2.5 ± 1.1	0.16 ± 0.02	0.18 ± 0.02	1.1 ± 0.5	11.8 ± 4.5	1.12 ± 0.05	Good

d_g_—geometric diameter; ρ—density; D_aero_—aerodynamic density; CI—Carr index; HR—Hausner ratio.

## Data Availability

The data presented in this study are available in RODBUK at https://doi.org/10.58032/AGH/PYCCPG.

## Abbreviations

The following abbreviations are used in this manuscript:

LRTIs	Lower respiratory tract infections
NLC	Nanostructured lipid carriers
COPD	Chronic obstructive pulmonary disease
DPI	Dry powder for inhalation
OPA	O-phthalaldehyde
LDH	Cytotoxicity detection kit
PBS	Phosphate-buffered saline
OLE	Oleic acid
EE	Encapsulation efficiency
DL	Drug loading
TOB	Tobramycin
SD	Standard deviation
CI	Carr index
HR	Hausner ratio
